# The role of resource orchestration in humanitarian operations: a COVID-19 case in the US healthcare

**DOI:** 10.1007/s10479-022-04963-2

**Published:** 2022-09-29

**Authors:** Konstantinos Baltas, Ranadeva Jayasekera, Gazi Salah Uddin, Thanos Papadopoulos

**Affiliations:** 1 Finance Group, Essex Business School, The University of Essex, Wivenhoe Park, Colchester, CO4 3SQ United Kingdom; 2 Trinity Business School, Trinity College, Dublin, Ireland; 3 Department of Management & Engineering, Linköping University, 581 83 Linköping, Sweden; 4 Kent Business School, University of Kent, Chatham Maritime Kent, ME4 4AG United Kingdom

**Keywords:** Resources, COVID-19, Orchestration, Pandemic, Healthcare operations

## Abstract

This paper investigates the role of resource allocation in alleviating the impact on from disruptions in healthcare operations. We draw on resource orchestration theory and analyse data stemming from US healthcare to discuss how the US healthcare system structured, bundled and reconfigured resources (i.e. number of hospital beds, and vaccines) during the COVID-19 pandemic. Following a comprehensive and robust econometric analysis of two key resources (i.e. hospital beds and vaccines), we discuss its effect on the outcomes of the pandemic measured in terms of confirmed cases and deaths, and draw insights on how the learning curve effect and other factors might influence in the efficient and effective control of the pandemic outcomes through the resource usage. Our contribution lies in revealing how different resources are orchestrated (‘structured’, ‘bundled’, and ‘leveraged’) to help planning responses to and dealing with the disruptions to create resilient humanitarian operations. Managerial implications, limitations and future research directions are also discussed.

## Introduction

Over the last years, operations and supply chains have been the subject of disruptions such as disease outbreaks (Queiroz et al., 2020; Sodhi, 2016) and physical catastrophes (Papadopoulos et al., 2017) which have negatively impacted on supply chains and operations and society (Craighead et al., 2007; Craighead et al., 2020; Ivanov, 2021; 2020; Thompson & Anderson, 2021) as well as vulnerable populations (Yagci Sokat & Altay, 2021). These include, for instance, disruptions in the automotive and electronics supply chain as a consequence of the Great East Japan earthquake and the tsunami in Thailand in 2011, which resulted in major losses for the manufacturers (e.g. Fujimoto and Park, 2013). While the field of operations and supply chains has been extensively investigated by research, the humanitarian aspects have been relatively neglected in the literature, with only a handful of dedicated special issues in the topic as well as one journal focusing on humanitarian operations and supply chain management (Behl and Dutta, 2018; Katsialaki et al., 2021).

The interest on humanitarian operations and supply chain management is an outcome of the rate of growth of both natural and man-made disruptions/disasters that may impact the existence of mankind (Behl and Dutta, 2018); amongst these, the recent focus on COVID-19 and the numerous studies and its implications in different fields (Flynn et al., 2021). The impact of COVID-19 pandemic on supply chains and operations has been the subject of many scholars and academic outlets (e.g. Choi, 2020; Chowdhury et al., 2021; Ivanov & Dolgui, 2020a, 2020b; Ivanov, 2021) amongst others. Reviews of the literature have looked into the impact of epidemics on logistics and supply chains (e.g. Queiroz et al., 2020) and panic buying during epidemics or pandemics (Yuen et al., 2020). In particular, researchers have investigated the ripple effects of COVID-19 on supply chains (e.g. Ivanov, 2020a, 2020b) and how simulation can help predict impacts (Ivanov & Das, 2020; Ivanov & Dolgui, 2020b; Ivanov, 2020a). Others have suggested different supply chain resilience strategies (e.g. Chen et al., 2019; Ivanov & Sokolov, 2019), or have highlighted the role of digitization (Ivanov et al., 2019) in dealing with the repercussions of the pandemic, or focused on production recovery planning in manufacturing for high-demand items (Paul and Chowdhury, 2020), or supply network resilience strategies (Azadegan & Dooley, 2021). There are also studies that have focused on multiple countries (E.g. Nikolopoulos et al., 2020) or continents due to the devastating impact of COVID-19, e.g. US (Lemke et al., 2020; Mehrotra et al., 2020; Sharma et al., 2020).

The COVID-19 pandemic has highlighted the need for humanitarian action to overcome the critical situation of vulnerable populations, inter alia (de Camargo Fiorini et al., *forthcoming*; Queiroz et al., 2020) and has underlined the importance of having appropriate resources and planning in place to deal with the pandemic. The appropriate allocation and management of resources is *sine qua non* to achieving operational success (Chen et al., 2017; Johansson & Olsson, 2017; Taleizadeh, 2018). Dolgui et al. (2018) argue for the appropriate allocation of limited resources in those circumstances where disasters lead to humanitarian challenges and industrial crises to balance both human life rescue and industrial sector recovery. Scholars (Behl and Dutta, 2018; Chiapetta Jabbour et al., 2019; Craighead et al., 2020) suggest that there is limited literature in terms of (i) how public operations and supply chains could be involved and organised so as to support the preparation and prevention of disasters; (ii) what kind of resources need to be (re-) configured to deal with different kinds and pace of disasters; and (iii) the utilisation of organisation theories in the area of humanitarian logistics and supply chain management. In a recent study, Ma et al. (2021) proposed a dynamic programming model that allocates hospital beds in three types of patients, that is, COVID-19, emergency, and elective-care. Still, limited studies focus in healthcare context as mathematical modelling or researchers’ opinions were the main method of investigation (Chowdhury et al., 2021).

This paper addresses these gaps, considering (i) the importance of resources in dealing the repercussions of the pandemic, but also to plan for future disruptions and (ii) the paucity of the literature in investigating how resources can be better orchestrated to plan for future waves of COVID-19 or similar pandemics and disruptions; and (iii) the need for theory-driven research on humanitarian supply chains (Chiappetta Jabbour et al., 2019; Dubey et al., 2019a, 2019b; Katsialaki et al., 2021), drawing on Bloomberg data. The sample period was from 15 May 2020 to 29 June 2021 (411 daily observations). For vaccine, the period started from 1 Jan 2021 to 29 June 2021 (180 daily observations). We employed a quantile regression technique to understand the behaviour of resources in the presence of the various waves associated with the pandemic in the United States. We use the rate of change (i.e. returns) of the variables in concern (i.e. change in estimated hospital beds for Covid patients (RHB), change in daily Covid related deaths (RDD), change in daily Covid positive cases (RCC) and the daily change in vaccination doses (RVV) administered to the population). Motivated by the argument of Tabaklar et al. (2015) on the need to use theories from other disciplines to advance the literature humanitarian supply chains, we use Resource Orchestration Theory (ROT) (Sirmon et al., 2007, 2011) that suggests that the focus should not be only on how a firm possesses valuable, rare, and difficult to substitute resources (Barney, 1991) but also on how managers ‘structure’, ‘bundle’, and ‘leverage’ resources to achieve value and acquire sustainable competitive advantage. This theory has been used recently by Ye et al. (2022) to investigate how digital technology assets across supply chains can help mitigate the negative impact of COVID-19 to operations. We use ROT to illustrate how the resources came together (i.e. were structured and bundled together) to create a ‘COVID-19 service capability’ and therefore allowed the system to deal with the repercussions of the pandemic.

The structure of the paper is as follows: Sect. 2 presents a brief review of the literature on operations and supply chain disruptions and focuses on COVID-9. Section 3 discusses our theoretical lens whereas Sect. 4 our methodology and estimations Our findings are presented in Sect. 5 while the theoretical and managerial implications in Sect. 6. Section 7 concludes the paper, providing limitations and future research directions.

## Disruptions in operations and supply chain management: A resource perspective

Over the last years scholars have highlighted the importance of supply chains for the economy and society, stating the importance of resilience (Christopher & Peck, 2004; Dubey et al., 2019a; Spiegler et al., 2012) and efficiency and transparency using digital technologies (Dubey et al., 2019b; Queiroz et al., 2020; Wamba et al., 2015; Wang et al., 2016).

In late 2019 the resilience and efficiency of supply chains and operations has been replaced by unprecedented shocks created by COVID-19 pandemic, which has had devastating disruptions and brought numerous challenges to operations, supply chains, and society in general (Choi, 2020; Ivanov & Dolgui, 2020a, 2020b; Sarkis et al., 2020). Altay and Green (2006, p. 475) argue that “disasters are large intractable problems that test the ability of communities and nations to effectively protect their populations and infrastructure, to reduce both human and property loss, and to rapidly recover”. It is noted that the SCM literature has analysed various types of disruptions brought by epidemics in the past including influenza, cholera, and malaria, *inter alia* (Queiroz et al., 2020). However, COVID-19 differs in terms of its global impact on supply chains as well as its unanticipated critical effects and consequences which will be carried over into the future (Flynn et al., 2021).

Dasaklis et al. (2012) argued that the literature on operations and supply chain management focusing on disruptions is devoted primarily to *resources and their allocation optimization*. Scholars (Chowdhury and Quaddus 2016) have stated the importance of resources after the disruption(s) to ensure response and recovery ability and develop dynamic capabilities, whereas in a recent study Nandi et al. (2020) looked at localization, agility and digitization capabilities and blockchain technology -related resources and capabilities to improve post COVID-19 supply chains. Ye et al. (2022) have investigated how firms orchestrate differently their digital technology resources (assets) to achieve better supply chain performance during COVID-19 disruption. Other scholars, however, state that research on the importance of resources on the development of capabilities and building supply chain resilience is still unexplored (Kähkönen et al., 2021; Ivanov, 2021). *Hence, there is a dearth of research on the role of resources to develop capabilities and improve operations/supply chain resilience.*

Approaches drawn from operations research and operations management in addressing the complex repercussions of disruptions (e.g. Besiou et al., 2018; Ivanov et al., 2017; Queiroz et al., 2020; Snyder et al., 2016) such as Markov chains, network theory (Hosseini and Ivanov 2019) simulation (Zhao et al., 2020; Ivanov, 2020a) have been used to discuss the role of resources within disruptions. Sawik (2019) has drawn on optimisation and developed a novel two-period modelling approach for supply chain disruption mitigation and recovery. Ivanov (2020a) has used simulation to discuss and predict the impact of epidemic outbreaks on supply chain performance drawing on COVID-19 and using simulation and optimization software. Their study provided useful lessons on how to predict short- and long- term impacts of epidemics and how managers could use such tools to change parameters (resource allocation) and maintain supply chain performance. Dubey et al., (2019a, 2019b) suggest that such methods have advantages in terms of addressing uncertainties associated with disaster location and demands as well as human actors’ coordination, demand forecasting and resource optimization in terms of mitigation, preparedness, response, and recovery. Nevertheless, there is criticism to these methods by scholars who claim that such methods fail to provide an in-depth understanding of the disaster relief field as well as how resources come together to address the disruption or plan for future/potential disruptions (Kovács and Spens 2011; Holguín-Veras et al., 2012). Furthermore, with the use of such methods there may be challenges in the disaster relief team to understand the needs of the stakeholders in need and the provision of sufficient relief operations (Altay, 2008; Gunasekaran et al., 2018).

Ivanov (2020a, 2020b) argues that attention should be on academics helping in establishing appropriate resilience measures to help companies survive and navigate through the pandemics (incl. COVID-19). To this extend, theories such as “resource-based view, dynamic capabilities, contingency theory can assist to frame empirically-grounded analytics and to examine the impacts of epidemic outbreaks” (Queiroz et al., 2020). However, in an earlier paper, Chowdhury and Quaddus (2017) argue against resource-based view or dynamic capabilities view as they “fail to identify processes, resources and paths that increase competencies during supply chain uncertainties” (Kähkönen et al., 2021, p.2). Furthermore, systematic reviews of the literature (Chowdhury et al., 2021; Queiroz et al., 2020) suggest a paucity in the literature discussing not only the effects of the pandemic (incl. COVID-19) on operations and supply chains but also in the application of theories to understand operations and supply chain behaviour before, during, and after a pandemic (Craighead et al., 2020). Craighead and colleagues draw on 10 theories such as resource dependency theory (Salancik & Pfeffer, 1978), institutional theory (DiMaggio & Powell, 1983), and resource orchestration theory (Sirmon et al., 2007) that could help researchers making sense of what happened during a disruption, how organizations responded, and how resources can be adjusted to render structures and processes resilient when/if other pandemic and disruption occurs. Such theories, Simon and colleagues believe, could also help practitioners by providing insights to decision makers when developing their plans to respond to disruptions. Therefore, *more research is needed on theories that can explain how organisations/supply chains handle resources during disruptions such as COVID-19* (Kähkönen et al., 2021; Queiroz et al., 2020; Yu et al., 2019).

This study draws on resource orchestration theory (Sirmon et al., 2007) to address the above research gaps. We investigate how resources were structured, bundled, and leveraged to deal with the repercussions of disruptions created by COVID-19 in the US healthcare system. Our focus on US healthcare system is justified by the number of confirmed COVID-19 cases and deaths, as well as on the devastating consequences and pressure of the coronavirus on hospital resources. A recent article by the FT suggests that “as coronavirus has swept across the US, it has ravaged the country’s healthcare system. Even with money from a $175bn bailout, many hospitals are facing critical cash shortages…”^1^ Furthermore, recent COVID-19 studies focusing on the US context have either used stochastic optimization model for allocating and sharing critical resources (Mehrotra et al., 2020), or have identified the strategies different companies use to deal with the pandemic relying on twitter data from NASDAQ 100 firms (Sharma et al., 2020), or the role of social networks of various supply chain players e.g. transportation providers, in rendering the supply chain more resilient to disruptions/pandemics. Still, these studies have not focused on healthcare processes and have not drawn upon theories that could further enhance our understanding of the disaster relief field as well as how resources come together for mitigation, preparedness, response, and recovery.

Resource orchestration theory is discussed in the next section.

## Resource orchestration theory and disruptions

Resource orchestration theory (ROT) (Sirmon et al., 2007, 2011) starts from the premise that a sole investigation of the resources a firm possesses does not provide a complete picture of its performance (Baert et al., 2016); it is how managers mobilise and leverage firm resources to achieve objectives that is also important. ROT builds on Barney’s (1991) argument that resources that are valuable, rare, and difficult to substitute provide sustainable competitive advantage; but the emphasis needs to be in how resources are orchestrated to develop and leverage capabilities. Literature (e.g. Simon et al., 2007; Gruber et al., 2010; Sirmon et al., 2011) has argued that in resource orchestration requires structuring the portfolio of resources, in terms of ‘structuring’ resources, that is, acquiring, accumulating, and divesting; ‘bundling’ -that is, integrating- resources to create capabilities, as well as ‘leveraging’ -that is, understanding the capabilities needed, coordinating the resources and deploying these resources to create capabilities- in the marketplace to create value (Sirmon et al., 2007). Although other theories, such as RBV and Dynamic Capabilities view have been used to investigate the role of resources (e.g. Arda et al., 2021; Chahal et al., 2020; Schilke et al., 2018). However, Sirmon et al. (2011) argue that these theories may explain how internal and external capabilities help organisations and supply chains respond to fast environmental changes such as disruptions, but they overlook the relationship between how the resources are acquired, bundled, and deployed. It is, then, not only how resources are chosen but how they are deployed and used efficiently (Sirmon et al., 2011), which is within the focus of this paper. D’ Oria et al. (2021) in their review and comparison of RBV and ROT argue that ROT moves from the importance of resources’ possession to “detailing resource-use processes and the importance of their synchronizing orchestration actions” (p. 1385) as it links resources, actions, and performance. ROT, therefore, does not displace RBV; on the contrary, it helps specify those resources and their orchestration processes to affect value and performance.

In operations and supply chain literature ROT has been used to analyse different types of product recalls and the ways firms endow resources and orchestrate activities around these resources (Ketchen et al., 2014); the ways companies use performance management systems to orchestrate their responses to organizational challenges and whether these uses positively affect operational, strategic, and external stakeholder related capabilities and performance over time (Koufteros et al., 2014); and how IT competency affects the relationship between supply chain integration and firm performance (Liu et al., 2017). Wong et al. (2018) have used ROT to study how internal, supplier, and customer sustainable development strategies can orchestrate different resources in the supply chain, and their impact on lean, green, and financial performance. Gong et al. (2018) focused on the impact of resource investments on profitability, as well as on what kind of resource configurations can lead to high profitability, whereas Burin et al. (2020) argued that ROT can help in understanding how ambidexterity can complement IT competences in developing supply chain flexibility. Kristoffersen et al. (2021) combined resource-based and resource-orchestration view to measure the business capability of firms for circular economy and the relationship amongst this capability, economy implementation, resource orchestration capability, and firm performance. In a recent study, Ye et al. (2022) used ROT to discuss the deployment of digital technology assets to achieve better supply chain performance during COVID-19 disruption. These studies highlight the importance of ROT in helping academics and managers understand (and ex ante predict) configurations of resources that lead to the achievement of competitive advantage. However, the theory is still underexplored within operations and supply chain research (Craighead et al., 2020).

In their review of theories related to pandemics and operations/supply chain research, Craighead et al (2020) suggest that ROT could provide an interesting lens with regards to: (i) outsourcing decisions to deal with diverse supply and demand; (ii) the long-term effects of ‘on-the-spot’ resource bundling have; and (iii) discussing how resource reconfiguration can result to different types of value during pandemics. Hence, within the operations studies related to COVID-19, we posit, following Craighead et al. (2020), that the theory can help in understanding the orchestration problems organizations and healthcare systems experience during the pandemic. Scholars could examine, for instance, how resources’ ‘structuring’, ‘bundling’, and ‘leveraging’ impact on creating flexibility/transiliency and deal with the longer implications of the crisis. Furthermore, firms would need to investigate how and what type of value they can create by bundling resources. Following the endorsement by Craighead et al. (2020), in this paper we use ROT to investigate how the US healthcare system has structured, bundled, and leveraged resources to provide value, that is, deal with COVID-19 and its repercussions. We outline our methodology in the following section.

## Methodology

The primary objective of this study is to gain an in-depth understanding of how two key resources (i.e. number of hospital beds, and vaccines) have been structured, bundled, and leveraged during COVID-19 pandemic. A great deal of empirical literature on the interdependence between resource orchestrations may have significant effect evidence of increased interdependence as a result of the COVID crisis. For this reason, we have hypothesized that the effects of the resource orchestration on the conditional return distribution may be significant and could differ across quantiles. Whether those effects are permanent or transitory is an empirical issue to be corroborated by the covid data. Throughout the paper we have emphasized that our analysis accounts for the impact of different explanatory variables on the quantiles of the conditional returns distribution of US healthcare system. Therefore, we employ a quantile regression technique to understand the behaviour of these resources in the presence of the various waves associated with the pandemic in the United States. We use the rate of change (i.e. returns) of the variables in concern (i.e. change in estimated hospital beds for Covid Patents (RHB), change in daily Covid related deaths (RDD), change in daily Covid positive cases (RCC) and the daily change in vaccination doses (RVV) administered to the population) as these variable are stationary and is a necessary prerequisite for our econometric analyses.

A common practice is to treat observed pattern of the movement of the variable/(s) in concern as an ‘information set’, which one uses to ‘predict’ as the next strategic response. But, this information set conceals and is unaccounted for the noisy signals arising out of, for instance, dynamic movements in other related fundamental drivers (representing parameter-driven sentimental values) associated with other interconnected factors. Eventually, a component of this ‘information set’ specific to a resource (i.e. estimated hospital beds for Covid patients), becomes a common component because noises generally display transmissive and transformative effects (Gillaizeau et al., 2019). The problem most often neglected is that whilst it is the entire dynamic path of a given resource and associated factors that determine the information set, inference is based only on the centre of the distribution. There are essentially two ways to understand cross-domain dynamic correlation: first, a systemic approach (such as estimation within a vector autoregression with/without long-memory), where interdependence across markets is assumed, but not modelled (Cheah et al., 2018). Yet, using this approach, one would be able to shed light on the ‘average’ dynamic effect, while being silent on what is happening on the other part of the distribution of this relationship. The second approach, which we propose in this paper, is a full-distributional approach where focus is laid on each part of the distribution of the variable; in our case, it is a study of a quantile-based dynamic causal structure at various parts of the distribution of the change in the estimated hospital beds. A theoretical expectation is that a dynamic relation between two variables in different domains, in A and B, for instance, will be heterogeneous over the entire range of the distribution. By modelling such a heterogeneity one would be able to gather complete information about the directional prediction pattern of one variable over the other at different parts of the distribution of the tail. A further implication is that since ‘fat tailed’ distributions depict implicit ‘herd behaviour’ synonymous with financial markets influenced by human biases (generated by asymmetric and incomplete information plus bounded rationality of agents), similar biases may be present in allocating assets; estimated hospital beds in our case. It is only when one can fully characterise the relationship of this ‘herd’ dynamics, it is possible to create an exhaustive information set that can be used to predict the dynamic path of one over the others. We model directional predictability across variables (i.e. rate of change in daily COVID-19 related deaths, change in daily COVID-19 positive cases and the daily change in vaccination doses administered to the population) over the entire distribution of the estimated hospital beds and appears to be the first study to propose a complete characterisation of tail dependence of a key scarce resource in the estimated hospital beds in the face of the Covid 19 pandemic in the United States.

### Estimations

We account for the impact of RDD, RCC AND RVV on RHB by considering the way in which the conditional τ quantile of the RHB distribution (${\text{y}}_{{\text{t}}}$), ${\text{Q}}_{{{\text{yt}}}} \left( {\tau \left| {{\text{y}}_{{{\text{t}} - 1}} ,{\text{d}}_{{\text{t}}} ,{\text{X}}_{{\text{t}}} ,{\text{X}}_{{{\text{t}} - 1}} } \right.} \right)$, is influenced by co-movement and causality effects of RDD, RCC and RVV. Hence:

1 $$ \begin{aligned} {\text{Q}}_{{{\text{yt}}}} \left( {\tau \left| {{\text{y}}_{{{\text{t}} - 1}} ,{\text{d}}_{{\text{t}}} ,{\text{X}}_{{\text{t}}} ,{\text{X}}_{{{\text{t}} - 1}} } \right.} \right) = & \,\alpha \left( \tau \right) + \beta \left( \tau \right){\text{y}}_{{{\text{t}} - 1}} \\ & + \gamma \left( \tau \right){\text{X}}_{{\text{t}}} + \eta \left( \tau \right){\text{X}}_{{{\text{t}} - 1}} \\ \end{aligned} $$

where y_t-1_ is the lagged value of the dependence variable. The parameters α(τ), β(τ) and ϕ(τ) account for the unconditional quantile, the effect of the lagged RHB and the impact of the RDD, RCC or RV, respectively. The parameter values in the (1xK) parameter vector γ(τ) for τ ∈ [0, 1] determine the structure of market co-movement between the dependent and (contemporaneous) explanatory variables, in such a way that (1) when γ(τ) values do not change across τ the dependence structure is constant, (2) when γ(τ) values increase (decrease) monotonically across τ the dependence structure increases (decreases), and (3) when γ(τ) values are similar (different) for high and low quantiles the dependence structure is symmetric (asymmetric) (see Baur, 2013; Mensi et al., 2014).

For a given τ, the parameters in Eq. (1) were estimated by minimizing the weighted absolute deviation as:

2 $$ \mathop {\arg K2\min }\limits_{\alpha \left( \tau \right),\beta \left( \tau \right),\phi \left( \tau \right),\gamma \left( \tau \right),\eta \left( \tau \right)} \sum\limits_{{{\text{t}} = 1}}^{{\text{T}}} {\rho_{\tau } \left( {y_{{\text{t}}} - \alpha \left( \tau \right) - \beta \left( \tau \right){\text{y}}_{{{\text{t}} - 1}} - \phi \left( \tau \right)d_{{\text{t}}} - \gamma \left( \tau \right){\text{X}}_{{\text{t}}} - \eta \left( \tau \right){\text{X}}_{{{\text{t}} - 1}} } \right),} $$

where $\rho_{\tau } \left( u \right) = u\left( {\tau - I\left( {u < 0} \right)} \right)$, 0 < τ < 1, and I(·) denotes the indication function. The problem in Eq. (2) was solved using the linear programming algorithm suggested by Koenker and D’Orey (1987). The pairs bootstrapping procedure proposed by Buchinsky (1995) was used to calculate standard error for the estimated parameters, given that this error is asymptotically valid under heteroskedasticity and misspecification of the QR function.

## Findings

### Data

Data was collected from Bloomberg. We adopted the following variables: (i) the estimated hospital beds (HB), the COVID-19 estimated patient impact and hospital capacity by State sourced from US Department of Health & Human Services; (ii) confirmed coronavirus death counts (DD); (iii) confirmed coronavirus case counts (CC); and (iv) vaccine administered (VV), which is the total cumulative number of COVID-19 vaccine doses administered. We utilized daily data and obtained log return respectively, named as RHB, RDD, RCC and RVV. The sample period is from 15 May 2020 to 29 June 2021 (411 daily observations). For vaccine, the period starts from 1 Jan 2021 to 29 June 2021 (180 daily observations). Table 1 presents the descriptive statistics of the variables in our sample (For more details on data, please see the Appendix).

**Table 1:** Descriptive statistics

Variables	HB	DD	CC	VV	RHB	RDD	RCC	RVV
Obs	411	411	411	180	411	411	411	180
Mean	59,468.24	339,000	1.71E + 07	1.60E + 08	*− 0.003*	0.005	0.008	0.026
Std. Dev	30,617.13	180,000	1.20E + 07	1.11E + 08	0.023	0.004	0.006	0.029
Min	15,942	87,559	1,443,188	3,489,090	− 0.093	0.000	0.000	0.000
Max	136,319	604,457	3.37E + 07	3.24E + 08	0.059	0.020	0.022	0.145
p5	19,987	108,211	1,872,660	7,867,504	− 0.039	0.001	0.000	0.003
p25	39,621	178,477	5,777,684	5.42E + 07	− 0.019	0.002	0.002	0.007
p50	47,799	282,268	1.49E + 07	1.52E + 08	− 0.004	0.004	0.007	0.017
p75	72,960	541,096	2.97E + 07	2.72E + 08	0.012	0.007	0.012	0.030
p95	125,702	598,744	3.34E + 07	3.18E + 08	0.035	0.011	0.017	0.086
Skew	0.977	0.225	0.116	0.054	0.021	0.999	0.367	2.637
Kurt	3.006	1.446	1.334	1.481	3.003	4.095	2.007	12.506

**Fig. 1:**
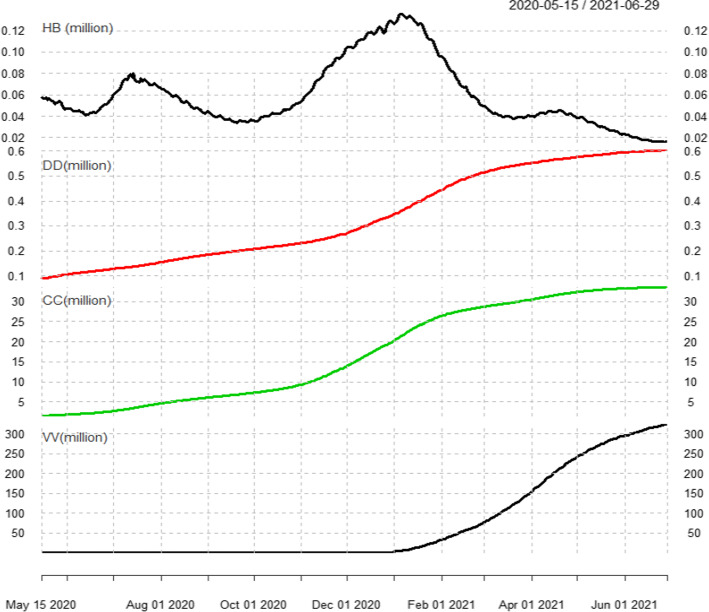
The dynamics of estimated hospital beds (HB), confirmed deaths (DD), confirmed cases (CC) and vaccine administered (VV)

The main variables are estimated hospital beds (HB), confirmed deaths (DD), confirmed cases (CC) and vaccine administered (VV), and the log return of estimated hospital beds (RHB), confirmed deaths (RDD), confirmed cases (RCC) and vaccine administered (RVV) respectively. The summary statistics includes the number of observations, mean, standard deviation, maximum, minimum, skewness, kurtosis, the percentiles (5% and 95%), median (50%), and quartiles (25% and 75%) distribution of the variables. Figure 1 plots the dynamics of estimated hospital beds (HB), confirmed deaths (DD), confirmed cases (CC) and vaccine administered (VV). We observe that the mean values of RDD, RCC and RVV are positive, with the minimum equal to zero, representing the beds, deaths and cases are accumulating by time. The minimum value of RHB is negative (-0.003), which means the beds can go upward or downward by times. From the quantile value and skewness, we observe right-skewed distribution for all variables. From the return distribution that shows a positive skew, one can expect recurrent slow growth and few rapid deteriorations of epidemic situation.

**Fig. 2:**
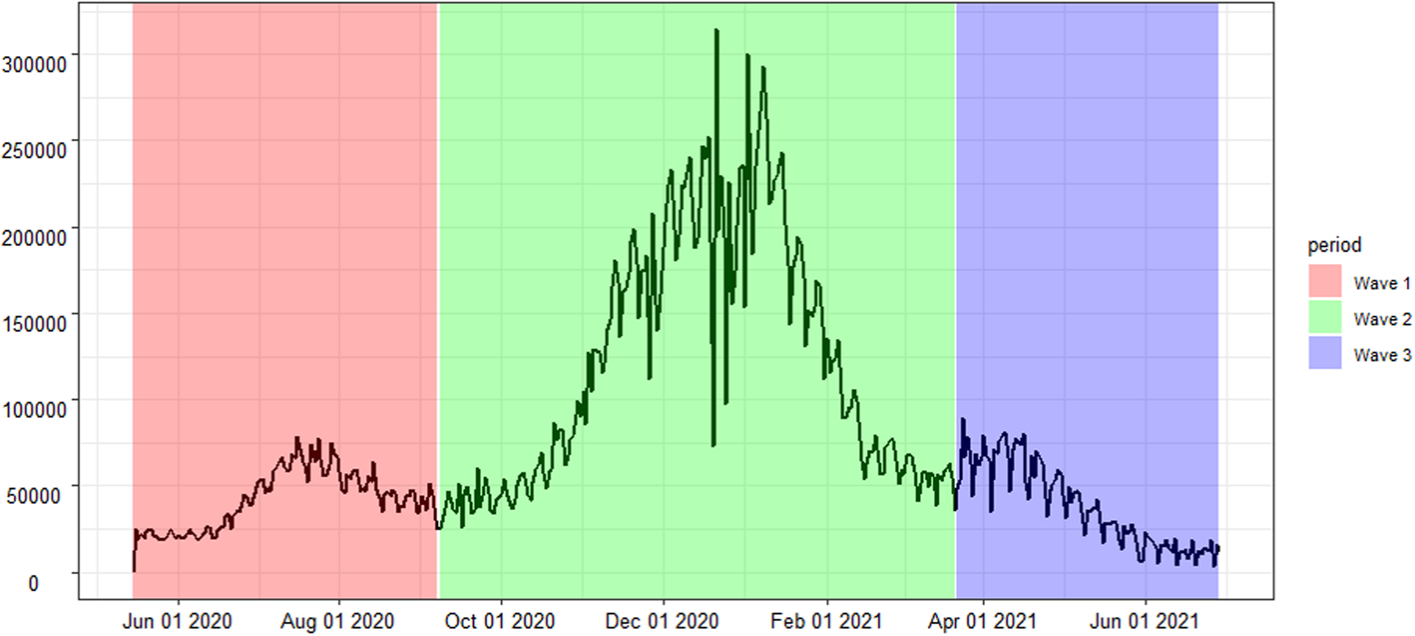
The three waves during the COVID-19 pandemic

By using the new cases confirmed every day, we recognize three waves for the epidemic, as shown in Fig. 2. We define the lowest point as the handover point of the two epidemic waves. The first wave is from 15 May 2020 to7 Sep 2020, the second wave is from 8 Sep 2020 to 21 Mar 2021, and the third wave is from 22 Mar 2021 to 29 Jun 2021. From Figs. 1 and 2, it can be observed that the estimated hospital beds and new cases daily confirmed have corresponding changes and similar trends as waves go by.

### Discussion of findings

We proceed firstly to draw some insight from 3-D graphs of the variables in concern and then conduct a more in-depth statistical analysis on the data to understand the dynamic behaviour of two key resources (i.e. the rate of change in the hospital beds and the rate of change in the covid vaccine administered to the population) in the face of the pandemic.

**Fig. 3:**
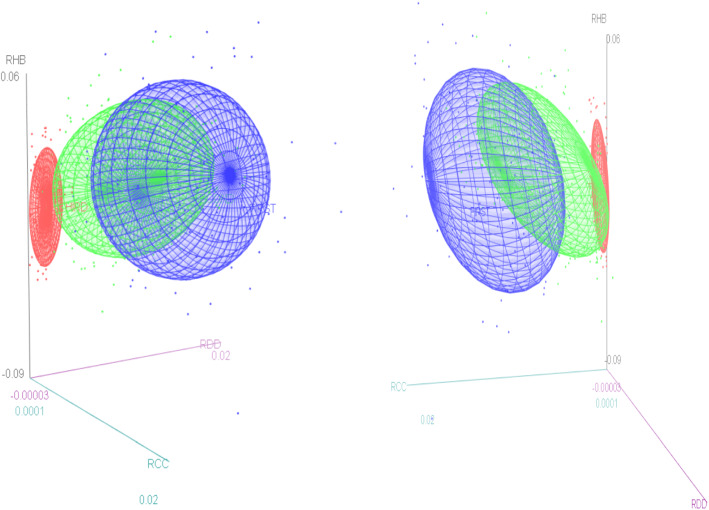
Rate of change in estimated hospital beds for Covid patients, in the presence of the change in daily Covid related deaths; and the rate of change in daily Covid positive cases over the different Covid waves

We observe (Fig. 3) that the ellipsoid related to the third wave is the leanest and the ellipsoid related to the first wave appears to be the fattest, whilst the ellipsoid related to the second wave demonstrates a transitional elongation. The length along the vertical line of a ellipsoid (i.e. the estimated hospital beds, i.e. Z axis) depicts the variation of the rate of change of the estimated hospital beds to a variation in the horizontal line (i.e. rate of change of daily covid deaths or the rate of change of daily confirmed covid cases), the x axis) whilst the remaining variable as the case may be (i.e. y axis) is held constant. Hence, we see that the rate of change of the estimated hospital beds is at its highest in response to a unit rate of change in the Covid deaths in the 3^rd^ wave, whilst it is at its lowest in the first wave, when the rate of change of the confirmed covid cases is held constant. Similarly, we see that the rate of change of the estimated hospital beds is at its highest in response to a unit rate of change in the Covid confirmed cases in the 3^rd^ wave whilst it is at its lowest in the first wave when the rate of change of the confirmed Covid deaths is held constant. These patterns suggests that the planning of the estimated hospital beds was most efficient as a response to the Covid deaths and Covid confirmed cases in the 3rd Covid wave whilst being least responsive in the first wave, perhaps hinting at the existence of an effective learning curve present when moving through the Covid waves. Furthermore, we see that the response of the hospital beds to the Covid confirmed cases is more pronounced when compared to the Covid related deaths in all 3 waves.

**Fig. 4:**
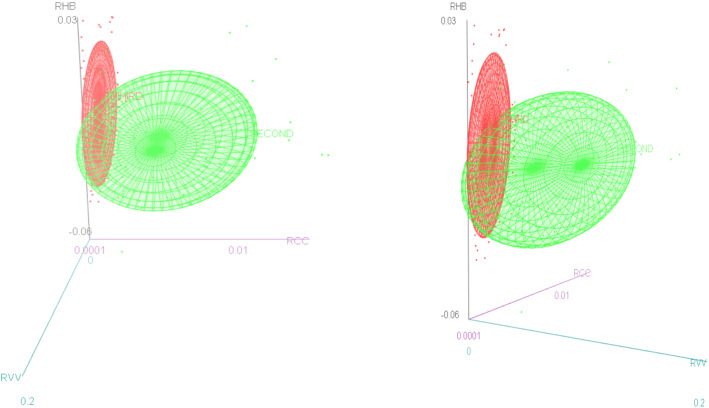
Change in estimated hospital beds for Covid Patents, in the presence of the change in daily Covid positive cases and the change in rate of the covid vaccine administered to the population over the different Covid waves

We follow a similar interpretation as above. Note that the first wave is not present as there were not publicly administered vaccines during the first wave. We see that the rate of change of the estimated hospital beds is at its highest in response to a unit rate of change in the Covid cases in the 3^rd^ wave whilst this ratio is much lower in the second wave, when the rate of change of the vaccines administered to the population is held constant. Similarly we see that the rate of change of the estimated hospital beds is at its highest in response to a unit rate of change in the vaccines administered to the population in the 3^rd^ wave whilst the ratio is much lower in the second wave, when the rate of change of the confirmed Covid cases is held constant. These patterns suggests that the planning of the estimated hospital beds was most efficient as a response to the Covid confirmed cases and was complimented well by the vaccine effect in the 3rd Covid wave (Fig. 4).

**Fig. 5:**
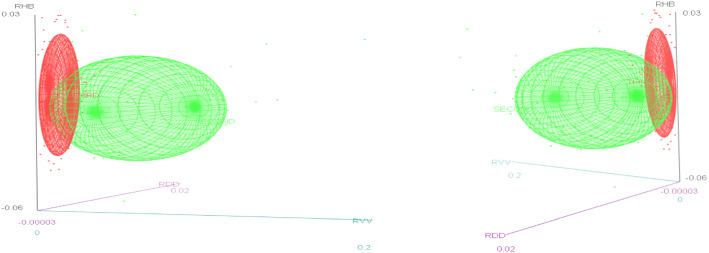
Change in estimated hospital beds for Covid Patents, in the presence of the change in daily Covid deaths and the change in rate of the covid vaccine administered to the population over the different Covid waves

We observe (Fig. 5) that the rate of change of the estimated hospital beds is at its highest in response to a unit rate of change in the Covid deaths in the 3rd wave whilst this ratio is much lower in the second wave, when the rate of change of the vaccines administered to the population is held constant. Similarly, we see that the rate of change of the estimated hospital beds is at its highest in response to a unit rate of change in the vaccines administered to the population in the 3^rd^ wave, whilst the ratio is much lower in the second wave, when the rate of change of the Covid deaths is held constant. These patterns suggests that the planning of the estimated hospital beds was most efficient as a response to the Covid deaths and was complimented well by the vaccine effect in the 3rd Covid wave.

We now proceed to discuss the findings and the interpretations of our econometric analysis using quantile regressions. As a first step we consider the results from the pair wise quantile regressions.

We first examine the effect of the rate of change of deaths (RDD) to the rate of change of hospital beds (RHB). The empirical evidence presented in Table 2 (see appendix) shows that RDD has a statistically significant negative influence in the determination of RHB especially at the higher levels of the distribution (i.e. at the 50% and 95% quantiles). This association is pronounced as seen with lags 1 and 28, and the lag length of RDD increases broadly across the higher quantiles of the distribution. Specifically, in lag 1 we notice that both the 50% and the 95% quantile exhibit a negative statistically significant correlation between the rate of change of deaths (RDD) and the rate of change of hospital beds (RHB) (there is a mild statistical significance at the lowest level of the distribution), while in lag28 and specifically at the 50% quantile we note the highest negative statistically significant correlation between them. These results indicate that especially over time the Covid related deaths have an effect of freeing up more beds in the RHB estimate.

**Table 2:** Statistical distributional properties of the considered variables

Variable	Obs	Mean	Std. Dev	Min	Max
RHB	411	− 0.003	0.023	− 0.093	0.059
RDD	411	0.005	0.004	0	0.02
RCC	411	0.008	0.006	0	0.022
RVV	180	0.026	0.029	0	0.205

We utilize daily data for the sample period from 2020-05-15 to 2021-06-29 (2087 daily observations)

As a next step, we consider the effect of the rate of change of confirmed cases (RCC) to RHB. Our findings in Table 3 (see appendix) suggest that RCC has a statistically significant positive influence in the determination of RHD especially at the higher levels of the distribution (i.e. at the 50% and 95% quantiles). Again, this association is pronounced in lag 1 and broadly across the higher quantiles of the distribution. To be more explicit, we notice that in all different lag orders (apart from lag 28) both the 50% and the 95% quantile exhibit a positive statistically significant correlation between the rate of change of confirmed cases (RCC) and the rate of change of hospital beds (RHB). Overall, these results indicate that especially over time, the change in confirmed Covid cases have an effect of absorbing up more beds in the RHB estimate.

**Table 3:** Dynamic relationship between RHB and RDD at 5%, 50% and 95% quantiles

	t	t − 1	t − 7	t − 14	t − 21	t − 28
${\text{RDD}}_{{\left( {0.05} \right)}}$	− 0.611	− 1.145*	− 1.156*	− 0.941*	− 0.819*	− 0.547
	(0.639)	(0.613)	(0.592)	(0.56)	(0.483)	(0.385)
Constant	− 0.037***	− 0.036***	− 0.035***	− 0.033***	− 0.034***	− 0.034***
	(0.002)	(0.003)	(0.003)	(0.003)	(0.003)	(0.002)
${\text{RDD}}_{{\left( {0.5} \right)}}$	− 0.81*	− 1.208***	− 0.865**	− 1.109**	− 0.724*	− 1.206***
	(0.449)	(0.367)	(0.35)	(0.478)	(0.39)	(0.338)
Constant	0	0.003	0	0.001	0	0.003
	(0.003)	(0.003)	(0.002)	(0.003)	(0.002)	(0.002)
${\text{RDD}}_{{\left( {0.95} \right)}}$	− 0.057	− 1.998**	− 0.055	− 0.007	− 0.005	0.018
	(1.223)	(0.84)	(1.05)	(1.182)	(1.405)	(0.963)
Constant	0.035***	0.041***	0.035***	0.035***	0.035***	0.035***
	(0.005)	(0.005)	(0.006)	(0.006)	(0.007)	(0.006)

^***^ p < .01, ** p < .05, * p < *.1*

Moreover, we are interested to investigate the impact of the rate of change in vaccines (RVV) administered to the population to the RHB. The results presented in Table 4 (see appendix) demonstrate that RVV has a statistically significant negative effect in the determination of RHD especially, once again, at the higher levels of the distribution (i.e. at the 50 and 95% quantiles). This association is pronounced with lags 14, 21 and 28 and broadly across the higher quantiles of the distribution. To be specific, the 25% quantile exhibits for all different lag orders a statistically insignificant correlation, whereas on the contrary, in both the 50 and the 95% quantile we report a high negative statistically significant correlation in the three highest lag orders between the rate of change in vaccines (RVV) administered to the population and the RHB. These results indicate that especially over time the increase in the vaccine administered to the population have an effect of freeing up more beds in the RHB estimate. It is interesting to note that the vaccine effect achieves statistical significance for the first time at lag 14 proving empirical validity for the implementation of the medical advice for a 14 day incubation period, post vaccine.

**Table 4:** Dynamic relationship between RHB and RCC at 5%, 50% and 95% quantiles

	t	t − 1	t − 7	t − 14	t − 21	t − 28
${\text{RCC}}_{{\left( {0.05} \right)}}$	0.186	0.284	− 0.031	− 0.083	− 0.11	− 0.178
	(0.499)	(0.515)	(0.524)	(0.488)	(0.41)	(0.391)
Constant	− 0.039***	− 0.04***	− 0.039***	− 0.038***	− 0.037***	− 0.037***
	(0.002)	(0.002)	(0.002)	(0.003)	(0.002)	(0.003)
${\text{RCC}}_{{\left( {0.5} \right)}}$	1.513***	1.338***	0.974***	0.587*	0.207	− 0.054
	(0.268)	(0.305)	(0.299)	(0.303)	(0.265)	(0.258)
Constant	− 0.015***	− 0.014***	− 0.012***	− 0.009***	− 0.006**	− 0.003
	(0.002)	(0.002)	(0.002)	(0.003)	(0.003)	(0.003)
${\text{RCC}}_{{\left( {0.95} \right)}}$	1.646***	1.446***	1.443***	0.901**	0.915**	0.761
	(0.333)	(0.371)	(0.434)	(0.45)	(0.445)	(0.836)
Constant	0.024***	0.026***	0.025***	0.029***	0.029***	0.029***
	(0.003)	(0.003)	(0.002)	(0.002)	(0.002)	(0.007)

^***^ p < .01, ** p < .05, * p < *.1*

### Wave Analysis

In the secondary stage we conduct a more granular analysis of our data. We examine the behaviour of our key resource RHB in the light of the other variables (RCC, RDD and RVV) in tandem, over the 3 COVID waves experienced in the United States. It is plausible (as we observe from our 3-d plots presented above) that the efficiency in the planning of the key resource RHB over the COVID waves were different, hence presenting a case for the granular level analysis across the different waves.

The empirical evidence of the wave analysis presented in Table 5 (see appendix) reveals that overall, there appears to be a significant negative association between RHB and RDD over the waves 1 and 2 across the whole distribution. This association is pronounced especially with wave 2 as we move to the highest level of the distribution. Specifically, during the second wave of the pandemic, we observe a strong negative statistically significant correlation between the rate of change of deaths (RDD) and the rate of change of hospital beds (RHB), in all three different quantiles and in all different lag orders. Interestingly, this correlation exhibits its highest value at 95% quartile across all different lag orders. However, it is interesting to note that in Wave 3, RDD becomes significant only at the extreme levels of the distribution (i.e. 5%, 95%) and this too only in contemporaneous terms and not all of the lags. These findings suggest that effect of the change in Covid deaths had little to no effect in estimating the hospital beds in the 3rd wave perhaps suggesting the learning effect gained in wave 1 and 2 in the allocation of this resource towards shifting the focus more towards treating the confirmed Covid cases than being influenced by the change in Covid deaths.

**Table 5:** Dynamic relationship between RHB and RVV at 5%, 50% and 95% quantiles

	t	t − 1	t − 7	t − 14	t − 21	t − 28
${\text{RVV}}_{{\left( {0.05} \right)}}$	0.062	0.092*	0.061	0.022	− 0.073	− 0.057
	(0.055)	(0.052)	(0.084)	(0.074)	(0.119)	(0.138)
Constant	− 0.041***	− 0.042***	− 0.04***	− 0.04***	− 0.036***	− 0.036***
	(0.002)	(0.002)	(0.003)	(0.003)	(0.003)	(0.004)
${\text{RVV}}_{{\left( {0.5} \right)}}$	0.016	0.035	− 0.028	− 0.175**	− 0.224***	− 0.182**
	(0.054)	(0.051)	(0.071)	(0.078)	(0.065)	(0.079)
Constant	− 0.012***	− 0.013***	− 0.012***	− 0.008**	− 0.007**	− 0.009**
	(0.002)	(0.002)	(0.003)	(0.003)	(0.003)	(0.003)
${\text{RVV}}_{{\left( {0.95} \right)}}$	− 0.07	0.012	− 0.121	− 0.203***	− 0.203**	− 0.253***
	(0.081)	(0.094)	(0.075)	(0.072)	(0.101)	(0.071)
Constant	0.028***	0.025***	0.027***	0.029***	0.029***	0.03***
	(0.005)	(0.005)	(0.005)	(0.005)	(0.005)	(0.005)

^***^ p < .01, ** p < .05, * p < *.1*

Next, we shift our focus to the association between RHB and RCC. Similarly, drawing on the aforementioned evidence, our results in Table 6 (see appendix) highlight a positive statistically significant association between those two variables over all waves across the lower to mid-level of the distribution. Once again, the results are more pronounced during the second wave of the pandemic. To be more explicit, our empirical evidence during wave 2 depicts always a statistically significant positive correlation between the rate of change of confirmed cases (RCC) and the rate of change of hospital beds (RHB), in both contemporaneous terms and in all different lag orders. However, what is interesting here is that in higher lags of RCC tends to lose some significance in wave 3. These results perhaps imply that the response in RHB was much faster and accurate in wave 3 (perhaps due to the learning effect) hence the lags did not have much of a correction to RHB. Furthermore, the fact that we see a negative significant association at a one month lag in wave 1, can be attributed to either a deficiency in the planning of the key resource, RHB, or to the fact that RHB might have reached an absolute maximum ceiling hence representing a practical bottleneck constraining this resource to move in tandem with the increasing daily Covid cases.

**Table 6:** Covid Wave Analysis: RHB Vs RDD at 5%, 50% and 95% quantile levels

	WAVE 1						WAVE 2					
	t	t − 1	t − 7	t − 14	t − 21	t − 28	t	t − 1	t − 7	t − 14	t − 21	t − 28
${\text{RDD}}_{{\left( {0.05} \right)}}$	− 2.768*	− 1.853	− 1.985	− 1.55	− 1.133	− 1.12	− 0.277	− 1.381	− 1.823	− 3.188***	− 3.939***	− 4.688***
	(1.598)	(1.15)	(1.374)	(1.465)	(2.14)	(1.941)	(0.848)	(1.126)	(1.198)	(1.209)	(1.231)	(1.397)
Constant	− 0.031***	− 0.034***	− 0.033***	− 0.035**	− 0.036**	− 0.035*	− 0.035***	− 0.027***	− 0.024***	− 0.013*	− 0.007	− 0.003
	(0.01)	(0.011)	(0.012)	(0.014)	(0.017)	(0.019)	(0.006)	(0.008)	(0.006)	(0.007)	(0.007)	(0.009)
${\text{RDD}}_{{\left( {0.5} \right)}}$	− 2.266***	− 3.257***	− 1.703*	− 1.313	− 0.25	0.679	− 1.647**	− 2.195***	− 2.292***	− 3.307***	− 4.065***	− 4.544***
	(0.805)	(0.864)	(1.013)	(1.039)	(0.669)	(1.171)	(0.7)	(0.491)	(0.606)	(0.843)	(1.009)	(0.979)
Constant	0.012*	0.018***	0.008	0.004	− 0.001	− 0.007	0.008*	0.013***	0.012***	0.017***	0.023***	0.026***
	(0.006)	(0.006)	(0.008)	(0.008)	(0.006)	(0.007)	(0.005)	(0.003)	(0.003)	(0.005)	(0.006)	(0.005)
${\text{RDD}}_{{\left( {0.95} \right)}}$	− 1.86	− 2.208***	− 1.596	− 1.106	− 0.789	− 0.368	− 2.281***	− 3.559***	− 3.175***	− 3.617***	− 3.972***	− 3.852***
	(1.679)	(0.84)	(1.379)	(1.628)	(2.021)	(2.207)	(0.752)	(0.752)	(0.609)	(0.55)	(0.726)	(0.786)
Constant	0.056***	0.057***	0.056***	0.054***	0.053***	0.052***	0.046***	0.05***	0.052***	0.054***	0.052***	0.051***
	(0.01)	(0.008)	(0.009)	(0.012)	(0.013)	(0.017)	(0.006)	(0.005)	(0.005)	(0.005)	(0.006)	(0.006)

	WAVE 3					
	t	t − 1	t − 7	t − 14	t − 21	t − 28
${\text{RDD}}_{{\left( {0.05} \right)}}$	11.723***	− 3.514	6.354	2.269	2.158	− 9.878
	(4.387)	(8.347)	(7.106)	(7.013)	(8.841)	(9.402)
Constant	− 0.051***	− 0.034***	− 0.044***	− 0.042***	− 0.042***	− 0.027**
	(0.006)	(0.009)	(0.008)	(0.009)	(0.011)	(0.012)
${\text{RDD}}_{{\left( {0.5} \right)}}$	4.158	− 4.492	1.894	− 0.684	− 3.033	− 3.772
	(3.08)	(6.021)	(3.207)	(4.684)	(3.636)	(3.129)
Constant	− 0.014***	− 0.005	− 0.013***	− 0.012*	− 0.012**	− 0.012**
	(0.004)	(0.008)	(0.005)	(0.006)	(0.005)	(0.005)
${\text{RDD}}_{{\left( {0.95} \right)}}$	− 5.747	− 6.801	− 8.2	− 11.116*	− 10.303**	− 10.882**
	(5.336)	(4.387)	(6.577)	(5.974)	(4.22)	(4.382)
Constant	0.032***	0.031***	0.034***	0.036***	0.034***	0.03***
	(0.006)	(0.005)	(0.006)	(0.006)	(0.008)	(0.007)

^***^ p < .01, ** p < .05, * p < *.1. The column name t, t − 1, …, t − 28 denote the time lag of RDD*

We now turn our attention, in examining, the behaviour of RHB in the presence of both RDD and RCC and the own lag of RHB simultaneously. As shown in Table 7 (see appendix), in contemporaneous terms the effect of all variables is prominent in wave 2 and 3 again suggesting an accurate learning effect. Furthermore, our empirical evidence suggests, that that as far as the lagged terms are concerned, all variables show a statistically significant effect on RHB in the mid-high (mostly) levels of the distribution, only in the second wave. We highlight here, that in line with our previous results, during the second wave of the crisis we observe the highest values of a strong negative (positive) statistically significant correlation between the rate of change of deaths (RDD) (confirmed cases (RCC)) and the rate of change of hospital beds (RHB). This is true, not only in both contemporaneous terms and in all different lag orders, but also, in all three 25, 75, 95% quantile. Moreover, it is interesting to note that the own lag of RHB becomes significant across all quantiles of the distribution in wave 3, implying a definite association in estimate of yesterdays beds to todays estimate, perhaps suggesting more faith in the reliability of the estimates in wave 3.

**Table 7:** Covid Wave Analysis: RHB Vs RCC at 5%, 50% and 95% quantile levels

	WAVE 1						WAVE 2					
	t	t − 1	t − 7	t − 14	t − 21	t − 28	t	t − 1	t − 7	t − 14	t − 21	t − 28
${\text{RCC}}_{{\left( {0.05} \right)}}$	− 0.402	− 0.327	− 0.465	− 2.29	− 3.53*	− 1.422	3.038***	2.789***	2.306***	0.957*	0.593	0.169
	(2.32)	(2.193)	(2.547)	(2.145)	(1.967)	(2.359)	(0.383)	(0.296)	(0.648)	(0.571)	(0.45)	(0.476)
Constant	− 0.044*	− 0.046*	− 0.043	− 0.017	0.007	− 0.022	− 0.052***	− 0.052***	− 0.05***	− 0.043***	− 0.042***	− 0.038***
	(0.025)	(0.025)	(0.03)	(0.026)	(0.028)	(0.04)	(0.004)	(0.004)	(0.005)	(0.004)	(0.004)	(0.005)
${\text{RCC}}_{{\left( {0.5} \right)}}$	0.96	1.133	0.1	− 1.808**	− 2.762***	− 3.78***	2.52***	2.021***	1.731***	1.537**	0.414	− 0.029
	(0.986)	(1.077)	(0.851)	(0.706)	(0.865)	(1.268)	(0.442)	(0.446)	(0.48)	(0.623)	(0.629)	(0.587)
Constant	− 0.017	− 0.02	− 0.006	0.023**	0.039***	0.056***	− 0.021***	− 0.018***	− 0.016***	− 0.013***	− 0.004	0
	(0.011)	(0.013)	(0.012)	(0.011)	(0.014)	(0.021)	(0.004)	(0.004)	(0.004)	(0.004)	(0.006)	(0.006)
${\text{RCC}}_{{\left( {0.95} \right)}}$	2.932***	3.266**	1.349	0.006	− 4.256**	− 5.418***	2.242	1.932	− 0.834	− 0.846	− 1.728**	− 2.393***
	(0.884)	(1.261)	(1.498)	(1.677)	(1.78)	(0.668)	(1.416)	(1.333)	(1.564)	(0.896)	(0.765)	(0.655)
Constant	0	− 0.002	0.027	0.049**	0.099***	0.111***	0.02*	0.022**	0.042***	0.043***	0.051***	0.057***
	(0.015)	(0.017)	(0.023)	(0.022)	(0.022)	(0.011)	(0.01)	(0.009)	(0.011)	(0.008)	(0.01)	(0.009)

	WAVE 3					
	t	t − 1	t − 7	t − 14	t − 21	t − 28
${\text{RCC}}_{{\left( {0.05} \right)}}$	11.613***	6.007	10.302***	6.548*	1.034	− 3.908
	(3.493)	(3.892)	(3.372)	(3.8)	(6.216)	(5.741)
Constant	− 0.049***	− 0.045***	− 0.05***	− 0.047***	− 0.041***	− 0.036***
	(0.005)	(0.005)	(0.005)	(0.006)	(0.009)	(0.009)
${\text{RCC}}_{{\left( {0.5} \right)}}$	6.611***	5.224**	4.318*	0.593	− 2.057	− 4.14
	(2.006)	(2.388)	(2.365)	(3.525)	(2.889)	(2.81)
Constant	− 0.018***	− 0.017***	− 0.016***	− 0.014***	− 0.012***	− 0.01**
	(0.004)	(0.004)	(0.004)	(0.004)	(0.004)	(0.005)
${\text{RCC}}_{{\left( {0.95} \right)}}$	6.67*	2.151	6.293	3.581	3.052	− 5.223
	(3.576)	(4.733)	(4.397)	(4.854)	(6.174)	(6.756)
Constant	0.015**	0.025***	0.016**	0.022**	0.02**	0.03***
	(0.007)	(0.006)	(0.008)	(0.009)	(0.009)	− 0.009

^***^ p < .01, ** p < .05, * p < *.1. The column name t, t − 1, …, t − 28 denote the time lag of RCC*

**Fig. 6:**
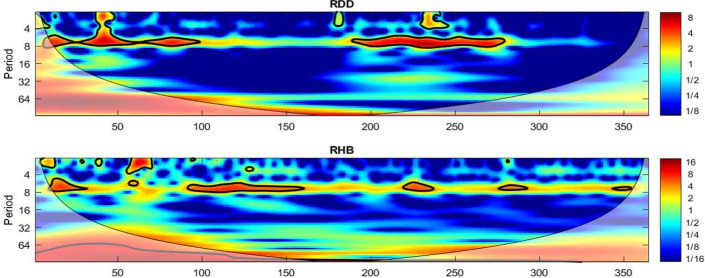
Wavelets

Finally, yet importantly, we present an interesting artefact using a wavelet power methodology (Fig. 6).

We observe extreme volatility around the 100–150 day period (which interestingly falls within wave 1) with the RHB, especially with its 4–8 day lag. Perhaps this suggests the chaotic nature in the estimation of the hospital beds in wave 1 and emphasizes that accuracy gained through the learning effect during wave 2 and 3.

Lastly, in order to account for any multicollinearity issue among our variables, we compute the variance inflation factor (VIF) diagnostics, one of the most widely-used diagnostics for multicollinearity. The variance inflation factor (VIF) is below 2.5 in all cases (Table 8), so we can safely conclude that the regression is free from any multicollinearity issues (Table 9).

**Table 8:** Covid Wave Analysis: RHB Vs RCC and RDD at 5%, 50% and 95% quantile levels

	WAVE 1						WAVE 2					
	t	t − 1	t − 7	t − 14	t − 21	t − 28	t	t − 1	t − 7	t − 14	t − 21	t − 28
${\text{RHB}}_{{{\text{t}} - 1\left( {0.05} \right)}}$	0.229	− 0.108	0.005	− 0.145	− 0.166	− 0.224	0.166	0.057	0.308**	0.349**	0.464***	0.529***
	(0.326)	(0.304)	(0.317)	(0.305)	(0.412)	(0.392)	(0.179)	(0.155)	(0.141)	(0.145)	(0.161)	(0.121)
${\text{RDD}}_{{\left( {0.05} \right)}}$	0.951	− 2.003	− 2.066	− 2.881	− 3.769	− 0.351	− 2.997***	− 4.911***	− 5.024***	− 4.307**	− 2.569	− 2.624
	(3.201)	(2.288)	(2.369)	(2.333)	(2.348)	(1.888)	(1.06)	(1.817)	(1.663)	(1.703)	(1.875)	(2.101)
${\text{RCC}}_{{\left( {0.05} \right)}}$	− 0.988	0.986	− 0.145	− 0.167	− 1.117	− 2.394	3.394***	3.947***	2.665***	2.07***	0.993	0.484
	(2.246)	(1.796)	(2.158)	(2.481)	(1.776)	(1.985)	(0.749)	(0.98)	(0.622)	(0.78)	(0.616)	(0.668)
Constant	− 0.04**	− 0.044**	− 0.031	− 0.022	− 0.004	− 0.006	− 0.033***	− 0.026***	− 0.016***	− 0.013*	− 0.016*	− 0.007
	(0.02)	(0.02)	(0.021)	(0.025)	(0.029)	(0.035)	(0.006)	(0.006)	(0.005)	(0.007)	(0.009)	(0.011)
${\text{RHB}}_{{{\text{t}} - 1\left( {0.5} \right)}}$	0.24**	0.146	0.327**	0.422***	0.305***	0.217*	0.493***	0.302***	0.509***	0.561***	0.528***	0.571***
	(0.116)	(0.109)	(0.135)	(0.122)	(0.111)	(0.121)	(0.101)	(0.094)	(0.11)	(0.075)	(0.058)	(0.053)
${\text{RDD}}_{{\left( {0.5} \right)}}$	− 3.258***	− 3.711***	− 1.286	− 2.129***	− 0.266	− 0.11	− 3.393***	− 4.588***	− 3.57***	− 3.806***	− 3.806***	− 3.494***
	(0.927)	(0.872)	(0.934)	(0.757)	(0.621)	(0.775)	(0.428)	(0.74)	(0.461)	(0.567)	(0.532)	(0.428)
${\text{RCC}}_{{\left( {0.5} \right)}}$	1.938**	2.199***	0.37	− 1.407*	− 2.491***	− 3.672***	1.944***	2.91***	1.929***	1.32***	1.185***	0.825***
	(0.756)	(0.686)	(0.904)	(0.794)	(0.636)	(1.151)	(0.384)	(0.565)	(0.589)	(0.456)	(0.39)	(0.276)
Constant	− 0.004	− 0.005	0.004	0.035***	0.04***	0.058***	0.003	0.001	0.004	0.01***	0.012***	0.013***
	(0.011)	(0.009)	(0.012)	(0.012)	(0.011)	(0.019)	(0.003)	(0.003)	(0.004)	(0.003)	(0.003)	(0.003)
${\text{RHB}}_{{{\text{t}} - 1\left( {0.95} \right)}}$	0.133	− 0.08	0.128	0.298	− 0.045	0.179	0.642***	0.435***	0.592***	0.589***	0.729***	0.612***
	(0.195)	(0.198)	(0.209)	(0.209)	(0.219)	(0.165)	(0.085)	(0.12)	(0.124)	(0.121)	(0.113)	(0.12)
${\text{RDD}}_{{\left( {0.95} \right)}}$	− 1.896*	− 3.368**	− 1.132	− 0.047	− 2.49	− 1.045	− 3.772***	− 3.798***	− 3.072***	− 3.736***	− 2.97***	− 2.538***
	(1.041)	(1.392)	(1.643)	(2.344)	(2.093)	(1.288)	(0.876)	(0.685)	(0.727)	(0.762)	(0.691)	(0.774)
${\text{RCC}}_{{\left( {0.95} \right)}}$	3.402***	2.985**	1.076	1.972	− 4.214***	− 4.906***	1.871***	1.895***	0.49	1.347	− 0.107	− 0.413
	(1.15)	(1.457)	(1.303)	(1.897)	(1.536)	(0.804)	(0.64)	(0.645)	(0.794)	(0.93)	(1.048)	(0.956)
Constant	0.004	0.02	0.036*	0.019	0.121***	0.111***	0.025***	0.028***	0.034***	0.032***	0.038***	0.037***
	(0.017)	(0.019)	(0.021)	(0.033)	(0.031)	(0.017)	(0.005)	(0.005)	(0.006)	(0.005)	(0.007)	(0.008)

	WAVE 3					
	t	t − 1	t − 7	t − 14	t − 21	t − 28
${\text{RHB}}_{{{\text{t}} - 1\left( {0.05} \right)}}$	0.653***	0.622***	0.553***	0.771***	0.634***	0.606***
	(0.116)	(0.198)	(0.131)	(0.169)	(0.156)	(0.187)
${\text{RDD}}_{{\left( {0.05} \right)}}$	− 7.244	− 7.382	− 16.748	− 13.398	− 19.643	− 16.241
	(14.507)	(12.077)	(12.229)	(13.471)	(13.956)	(10.099)
${\text{RCC}}_{{\left( {0.05} \right)}}$	8.206	7.415	12.001**	5.147	5.752	1.742
	(7.109)	(6.818)	(5.669)	(5.306)	(7.101)	(6.154)
Constant	− 0.025***	− 0.026***	− 0.023**	− 0.016	− 0.011	− 0.008
	(0.009)	(0.007)	(0.01)	(0.013)	(0.012)	(0.009)
${\text{RHB}}_{{{\text{t}} - 1\left( {0.5} \right)}}$	0.534***	0.413***	0.63***	0.615***	0.501***	0.526***
	(0.113)	(0.136)	(0.116)	(0.098)	(0.116)	(0.124)
${\text{RDD}}_{{\left( {0.5} \right)}}$	− 24.298***	− 13.79	− 22.383**	− 20.079**	− 17.073*	− 14.073
	(8.101)	(11.66)	(9.969)	(8.344)	(8.603)	(9.244)
${\text{RCC}}_{{\left( {0.5} \right)}}$	15.835***	8.52	12.944**	9.166*	4.925	3.176
	(4.885)	(7.279)	(5.971)	(5.343)	(5.414)	(6.193)
Constant	0.003	− 0.001	0.006	0.007	0.007	0.007
	(0.005)	(0.007)	(0.007)	(0.006)	(0.007)	(0.007)
${\text{RHB}}_{{{\text{t}} - 1\left( {0.95} \right)}}$	0.63***	0.407**	0.36	0.468**	0.385	0.453
	(0.208)	(0.18)	(0.233)	(0.19)	(0.264)	(0.283)
${\text{RDD}}_{{\left( {0.95} \right)}}$	− 9.133	− 6.239	− 9.463*	− 11.345*	− 10.806	− 11.654*
	(6.337)	(8.995)	(5.441)	(5.809)	(6.697)	(6.658)
${\text{RCC}}_{{\left( {0.95} \right)}}$	− 1.852	− 1.114	2.271	2.5	6.533	− 1.348
	(6.909)	(7.229)	(6.567)	(5.601)	(7.246)	(7.405)
Constant	0.034***	0.029***	0.031***	0.031***	0.024*	0.037***
	(0.008)	(0.006)	(0.011)	(0.008)	(0.014)	(0.009)

*** p < .01, ** p < .05, * p < .1*. The column name t, t− 1, …, t− 28 denote the time lag of RDD and RCC*

**Table 9:** VIF Test: RHB Vs RCC and RDD

	WAVE 1						WAVE 2					
	t	t − 1	t − 7	t − 14	t − 21	t − 28	t	t − 1	t − 7	t − 14	t − 21	t − 28
RHB_lag1	1.104	1.16	1.026	1.016	1.083	1.184	1.491	1.889	1.356	1.235	1.175	1.11
RDD	1.066	1.15	1.045	1.028	1.017	1.049	1.351	1.847	1.356	1.344	1.333	1.288
RCC	1.162	1.165	1.06	1.026	1.077	1.145	1.905	2.338	1.691	1.462	1.315	1.202

	WAVE 3					
	t	t − 1	t − 7	t − 14	t − 21	t − 28
RHB_lag1	1.318	1.259	1.2	1.142	1.084	1.115
RDD	2.178	2.353	2.035	1.916	1.789	1.793
RCC	2.28	2.648	2.081	1.898	1.703	1.655

*The column name t, t − 1, …, t − 28 denote the time lag of RDD and RCC*

## Theoretical and managerial implications

### Theoretical implications

The contribution of this paper is twofold:

(i) The paper contributes to the literature on disruptions (e.g. Hosseini and Ivanov 2019; Ivanov, 2020a, 2020b; Zhao et al., 2020; Ivanov, 2020a) by illustrating the role of appropriate resource allocation and (Dasaklis et al., 2012) during the pandemic. It argues for the importance of ‘structuring’, ‘bundling’, and ‘leveraging’ resources (Sirmon et al., 2007) (i.e. number of hospital beds, and vaccines) to create resilience in hospitals through appropriate resource allocation, assisting thereby decision makers in taking appropriate resource allocation decisions, and ensure fair deployment of resources during the pandemic. Our findings show that the rate of estimated hospital beds and change in vaccination doses have a statistically significant negative influence in the determination of rate of hospital bed usage while the rate of COVID cases demonstrates a statically significant positive influence. Interestingly, during the third wave the drop in COVID related deaths did not translate into learning in terms of hospital bed estimation. This may mean that the learning gained in the previous two waves shifted the focus into the treatment rather than being influenced by the change in Covid deaths. Our paper compliments recent research on dynamic resource allocation of resources during the COVID-19 pandemic (Ma et al., 2021). Our aim was not to offer a dynamic programming model to study the allocation of isolation and ordinary beds for patients, COVID-19, emergency, and elective care, but to illustrate how resources can be ‘structured’, ‘bundled’ and ‘leveraged’ to ensure resource allocation decisions and fair deployment of resources during the pandemic.
(ii) This paper illustrates the use of ROT, following the endorsement by Craighead et al. (2020) to use theories to explain resource allocation during the COVID-19 pandemic. It is argued that the use of ROT further adds to the literature on disruptions (Dubey et al., 2019a, 2019b; Kähkönen et al., 2021; Queiroz et al., 2020; Yu et al., 2019) as it goes beyond the importance of possessing resources to linking and deploying resources to achieve performance (Sirmon et al., 2007, 2011; D’ Oria et al., 2021; Ye et al., 2022) (and in our case appropriate resource allocation for decision making and hence dealing with the repercussions of COVID-19 for hospital operations). Hence it offers an alternative lens to those studies using e.g. RBV to study operations and supply chain disruptions, contributing thereby to the need for theory-driven research on humanitarian supply chains (Dubey et al., 2019a, 2019b).

### Managerial and policy implications

The results of this study can provide useful lessons to managers and policy makers on how to deploy and orchestrate (combine, that is) resources to deal with the repercussions of COVID. In particular, the bundling of resources such as number of beds and vaccine administrations can have an impact on the long-term existence of COVID and its unpredictable scaling and on the way the healthcare system can deal with unpredictable demand and disturbances in supply of healthcare and infrastructure. Having the appropriate resources in place and understanding how these can come together can help managers (re-) allocating resources when needed to deal with the repercussions of COVID. The following insights are offered:

Insight 1: it is important to orchestrate and predict the use of hospital beds in response to future covid confirmed cases and deaths.

Insight 2: it is important to orchestrate and predict the use of hospital beds in conjunction with vaccines’ administration in response to future covid confirmed cases and deaths.

Insight 3: it is important to understand the strength of the learning effect in response to the pandemic both from a managerial and policy standpoint when formulating strategies.

These insights can help policy/decision makers in devising appropriate resource allocation mechanisms and decision support systems to ensure fair distribution or resources during disruptions and pandemics so that the healthcare (hospital) systems are able to provide fast and efficient care to all patients.

## Conclusions

The study focused on how the orchestration of resources can lead to better resource allocation by decision makers during disruptions, drawing on the COVID-19 case within the operations of the US healthcare system. We investigated how the rate of hospital beds and vaccines could help, if bundled together, in dealing with the rate of COVID related deaths and cases. We drew on ROT as our focus was not on the possession of resources only, but how they come together to create resilience decision making capabilities. Our findings suggested that the rate of estimated hospital beds and change in vaccination doses have a statistically significant negative influence in the determination of rate of hospital bed usage while the rate of COVID cases demonstrates a statically significant positive influence. Interestingly, during the third wave the drop in COVID related deaths did not translate into learning in terms of hospital bed estimation.

The study has the following limitations. The resources used were related to the number of hospital beds and vaccinations. There are other resources also that can be used and bundled, however, the choice of these two was based on being the most important ones in the fight against the pandemic. Furthermore, there are significant challenges related to the acquisition, cleansing, and analysis of hospital data related to COVID-19. At the time of the study there were no data available regarding COVID-19 medication. It is also important to recognise the limitations and constraints of our findings especially when used to formulate managerial and policy level strategies. Whilst we have carried out stringent robustness checks to validate our results one needs to recognise that the impact of the pandemic can be different in different geographic areas and at different time scales. Furthermore, given the data availability we have limited our analysis to the key recourses and the study can be expanded by considering more resources. Furthermore, important factors such as the sentiment of public towards the pandemic needs to be considered (if data is available) as this could have a bearing on the effectiveness lockdown strategies and its knock-on effect on Covid contraction and ultimate Covid related deaths.

Future research could (i) focus on differences between public and private hospitals in terms of how resources could be orchestrated (ii) develop hypotheses based on ROT which could then be tested through e.g. surveys with managers (iii) compare resource orchestration for COVID-19 vs other patients with serious conditions (iv) study inpatients vs outpatients with COVID-19 and resource allocation (v) draw on management science to create models based on e.g. dynamic programming to study the allocation of resources (vi) use other types of resources including e.g. technology (ventilators) and other variables (e.g. medication prescribed) based on available data as well as considering the different characteristics of hospitals such as triage and capacity.

## Variable description

### Hcovcwhr Index—estimated hospital beds

COVID-19 Estimated Patient Impact and Hospital Capacity by State sourced from US Department of Health & Human Services: https://beta.healthdata.gov/dataset/COVID-19-Estimated-Inpatient-Beds-Occupied-by-COVI/py8k-j5rq.

## Ncovusde Index-confirmed deaths

Confirmed coronavirus (2019-nCov) death counts compiled by Bloomberg Newsroom. Counts are subject to change as governments survey and confirm cases. Data are based on reported values as of Midnight EST. Sources include Johns Hopkins University, World Health Organization, DXY, NHC, BNO News, China CDC, European CDC, US CDC, Italy Ministry of Health, Hong Kong Department of Health, Macau Government, Taiwan CDC, Government of Canada, Australia Government Department of Health, and Ministry of Health Singapore.

## Ncovusca index—confirmed cases

Confirmed coronavirus (2019-nCov) case counts compiled by Bloomberg Newsroom. Counts are subject to change as governments survey and confirm cases. Data are based on reported values as of Midnight EST. Sources include Johns Hopkins University, World Health Organization, DXY, NHC, BNO News, China CDC, European CDC, US CDC, Italy Ministry of Health, Hong Kong Department of Health, Macau Government, Taiwan CDC, Government of Canada, Australia Government Department of Health, and Ministry of Health Singapore.

## Ncovusva index -vaccine administered

Total cumulative number of COVID-19 vaccine doses administered. Data is sourced from {https://coronavirus.jhu.edu/vaccines}.

## Footnotes

1 https://www.ft.com/content/3bbb4f7c-890e-11ea-a01c-a28a3e3fbd33 (Accessed 19th July 2021).

## References

[CR1] Altay N, Gad-el-Hak M (2008). Issues in disaster relief logistics. Large-scale disasters: prediction control and mitigation.

[CR2] Altay N, Green W (2006). OR/MS research in disaster operations management. European Journal of Operational Research.

[CR3] Arda OA, Montabon F, Tatoglu E, Golgeci I, Zaim S (2021). Toward a holistic understanding of sustainability in corporations: resource-based view of sustainable supply chain management. Supply Chain Management.

[CR4] Azadegan A, Dooley K (2021). A typology of supply network resilience strategies: complex collaborations in a complex world. Journal of Supply Chain Management.

[CR5] Baert C, Meuleman M, Debruyne M, Wright M (2016). Portfolio entrepreneurship and resource orchestration. Strategic Entrepreneurship Journal.

[CR6] Barney J (1991). Firm resources and sustained competitive advantage. Journal of Management.

[CR7] Baur D (2013). The structure and degree of dependence: A quantile regression approach. Journal of Banking and Finance.

[CR8] Behl A, Dutta P (2018). Humanitarian supply chain management: A thematic literature review and future directions of research. Annals of Operations Research.

[CR9] Besiou M, Pedraza-Martinez AJ, Van Wassenhove LN (2018). OR applied to humanitarian operations. European Journal of Operational Research.

[CR10] Buchinsky M (1995). Estimating the asymptotic covariance matrix for quantile regression models: a Monte Carlo study. Journal of Econometrics.

[CR11] Burin ARG, Perez-Arostegui MN, LlorensMontes J (2020). Ambidexterity and IT competence can improve supply chain flexibility? A resource orchestration approach. Journal of Purchasing and Supply Management.

[CR12] Chahal H, Gupta M, Bhan N, Cheng TC (2020). Operations management research grounded in the resource-based view: a meta-analysis. International Journal of Production Economics.

[CR13] Cheah J-H, Sarstedt M, Ringle CM, Ramayah T, Ting H (2018). Convergent validity assessment of formatively measured constructs in PLS-SEM: on using single-item versus multiitem measures in redundancy analyses. International Journal of Contemporary Hospitality Management.

[CR14] Chen HY, Das A, Ivanov D (2019). Building resilience and managing post-disruption supply chain recovery: lessons from the information and communication technology industry. International Journal of Information Management.

[CR15] Chen J, Liang L, Yao DQ (2017). Pre-positioning of relief inventories for non-profit organizations: a newsvendor approach. Annals of Operations Research.

[CR16] Choi T-M (2020). Risk analysis in logistics systems: a research agenda during and after the COVID-19 pandemic. Transportation Research Part e: Logistics and Transportation Review.

[CR17] Chowdhury P, Paul SK, Kaisar S, Moktadir MA (2021). COVID-19 pandemic related supply chain studies: A systematic review. Transportation Research Part e: Logistics and Transportation Review.

[CR18] Christopher M, Peck H (2004). Building the resilient supply chain. International Journal of Logistics Management.

[CR19] Craighead CW, Blackhurst J, Rungtusanatham MJ, Handfield RB (2007). The severity of supply chain disruptions: Design characteristics and mitigation capabilities. Decisions Sciences.

[CR20] Craighead CW, Ketchen DJ, Darby JL (2020). Pandemics and supply chain management research: toward a theoretical toolbox. Decision Sciences.

[CR21] D’Oria L, Crook R, Ketchen D, Sirmon D, Wright M (2021). The evolution of resource-based inquiry: a review and meta-analytic integration of the strategic resources–actions–performance pathway. Journal of Management.

[CR22] Dasaklis TK, Pappis CP, Rachaniotis NP (2012). Epidemics control and logistics operations: a review. International Journal of Production Economics.

[CR23] DiMaggio PJ, Powell WW (1983). The iron cage revisited: institutional isomorphism and collective rationality in organizational fields. American Sociological Review.

[CR25] Dubey R, Gunasekaran A, Child SJ, Roubaud D, Wamba SF, Giannakis M, Foropon C (2019). Big data analytics and organizational culture as complements to swift trust and collaborative performance in the humanitarian supply chain. International Journal of Production Economics.

[CR26] Dubey R, Gunasekaran A, Childe S, Blome C, Papadopoulos T (2019). Big Data and Predictive Analytics and Manufacturing Performance: Integrating Institutional Theory, Resource-Based View and Big Data Culture. British Journal of Management.

[CR27] Flynn B, Cantor D, Pagell D, Dooley KJ, Azadegan A (2021). From the editors: introduction to managing supply chains beyond covid-19 - preparing for the next global mega- disruption. Journal of Supply Chain Management.

[CR28] Gillaizeau M, Jayasekera R, Maaitah A, Mishra T, Parhi M, Volokitina E (2019). Giver and the receiver: understanding spillover eects and predictive power in cross-market Bitcoin prices. International Review of Financial Analysis.

[CR29] Gong Y, Jia F, Brown S, Koh L (2018). Supply chain learning of sustainability in multi-tier supply chains. International Journal of Operations & Production Management.

[CR30] Gruber M, Heinemann F, Brettel M, Hungeling S (2010). Configurations of resources and capabilities and their performance implications: an exploratory study on technology ventures. Strategic Management Journal.

[CR31] Gunasekaran A, Dubey R, Fosso Wamba S, Papadopoulos T, Hazen B, Ngai EWT (2018). Bridging humanitarian operations management and organizational theory. Editorial. International Journal of Production Research.

[CR33] Holguín-Veras J, Hart WH, Jaller M, Van Wassenhove LN, Pérez N, Wachtendorf T (2012). On the unique features of post-disaster humanitarian logistics. Journal of Operations Management.

[CR34] Hosseini S, Ivanov D, Dolgui A (2019). review of quantitative methods for supply chain resilience analysis. Transportation Research: Part E.

[CR36] Ivanov D (2020). Predicting the impacts of epidemic outbreaks on global supply chains: a simulationbased analysis on the coronavirus outbreak. Transportation Research Part E.

[CR37] Ivanov D (2020). Viable supply chain model: Integrating agility, resilience, and sustainability perspectives. Lessons from and thinking beyond the COVID-19 pandemic. Annals of Operations Research.

[CR38] Ivanov D, Das A (2020). Coronavirus (COVID-19/SARS-CoV-2) and supply chain resilience: a research note. International Journal of Integrated Supply Management.

[CR39] Ivanov D, Dolgui A (2020). A digital supply chain twin for managing the disruptions risks and resilience in the era of Industry 4.0. Production Planning and Control.

[CR40] Ivanov D, Dolgui A (2020). Viability of intertwined supply networks: extending the supply chain resilience angles towards survivability. A position paper motivated by COVID-19 outbreak. International Journal of Production Research.

[CR42] Ivanov D, Dolgui A, Sokolov B (2019). The impact of digital technology and industry 4.0 on the ripple effect and supply chain risk analytics. International Journal of Production Research.

[CR43] Ivanov D, Pavlov A, Pavlov D, Sokolov B (2017). Minimization of disruption-related return fows in the supply chain. International Journal of Production Economics.

[CR44] Ivanov D, Sokolov B (2019). Simultaneous structural-operational control of supply chain dynamics and resilience. Annals of Operations Research.

[CR46] Johansson L, Olsson F (2017). Quantifying sustainable control of inventory systems with non-linear backorder costs. Annals of Operations Research.

[CR47] Katsaliaki K, Galetsi P, Kumar S (2021). Supply chain disruptions and resilience: a major review and future research agenda. Annals of Operations Research.

[CR48] Ketchen DJ, Wowak K, Craighead CW (2014). Resource gaps and resource orchestration shortfalls in supply chain management: The case of product recalls. Journal of Supply Chain Management.

[CR51] Koufteros X, Verghese AJ, Lucianetti L (2014). The effect of performance measurement systems on firm performance: a cross-sectional and a longitudinal study. Journal of Operations Management.

[CR52] Kovacs G, Spens KM (2011). Humanitarian logistics and supply chain management: the start of a new journal. Journal of Humanitarian Logistics and Supply Chain Management.

[CR53] Kristoffersen E, Mikalef P, Blomsma F, Li J (2021). The effects of business analytics capability on circular economy implementation, resource orchestration capability, and firm performance. International Journal of Production Economics.

[CR54] Lemke MK, Apostolopoulos Y, Sönmez S (2020). Syndemic frameworks to understand the effects of COVID-19 on commercial driver stress, health, and safety. Journal of Transport and Health.

[CR55] Liu CL, Kuo CS, Taih CL, Kee HL, Lun HYV (2017). Supply chain resilience, firm performance, and management policies in the liner shipping industry. Transportation Research Part a: Policy and Practice.

[CR56] Ma X, Zhao X, Guo P (2021). Cope with the COVID-19 pandemic: dynamic bed allocation and patient subsidization in a public healthcare system. International Journal of Production Economics.

[CR57] Mehrotra S, Rahimian H, Barah M, Luo F, Schantz K (2020). A model of supply-chain decisions for resource sharing with an application to ventilator allocation to combat COVID-19. Naval Research Logistics.

[CR58] Mensi W, Hammoudeh S, Reboredo JC, Nguyen DK (2014). Do global factors impact BRICS stockmarkets? A quantile regression approach. Emerging Markets Review.

[CR59] Papadopoulos T, Gunasekaran A, Dubey R, Altay N, Childe S, Fosso-Wamba S (2017). The role of big data in explaining disaster resilience for sustainability. Journal of Cleaner Production.

[CR60] Paul SK, Chowdhury P (2020). A production recovery plan in manufacturing supply chains for a high-demand item during COVID-19. International Journal of Physical Distribution & Logistics Management.

[CR61] Queiroz MM, Ivanov D, Dolgui A, Fosso Wamba S (2020). Impacts of epidemic outbreaks on supply chains: mapping a research agenda amid the COVID-19 pandemic through a structured literature review. Annals of Operations Research.

[CR62] Salancik GR, Pfeffer J (1978). A social information processing approach to job attitudes and task design. Administrative Science Quarterly.

[CR63] Sarkis J, Cohen MJ, Dewick P, Schröder P (2020). A brave new world: lessons from the COVID-19 pandemic for transitioning to sustainable supply and production. Resources, Conservation and Recycling.

[CR64] Sawik T (2019). Two-period versus multi-period model for supply chain disruption management. International Journal of Production Research.

[CR65] Schilke O, Hu S, Helfat CE (2018). Quo vadis, dynamic capabilities? A contentanalytic review of the current state of knowledge and recommendations for future research. Academy of Management Annals.

[CR66] Sharma A, Adhikary A, Borah SB (2020). COVID-19’s impact on supply chain decisions: strategic insights from NASDAQ 100 firms using twitter data. Journal of Business Research.

[CR67] Sirmon DG, Hitt MA, Ireland RD (2007). Managing firm resources in dynamic environments to create value: looking inside the black box. Academy of Management Review.

[CR68] Sirmon D, Hitt MA, Ireland RD, Gilbert BA (2011). Resource orchestration to create competitive advantage: Breadth, depth, and life cycle effects. Journal of Management.

[CR69] Snyder LV, Atan Z, Peng P, Rong Y, Schmitt AJ, Sinsoysal B (2016). OR/MS models for supply chain disruptions: a review. IIE Transactions.

[CR70] Sodhi MMS (2016). Natural disasters, the economy and population vulnerability as a vicious cycle with exogenous hazards. Journal of Operations Management.

[CR71] Spiegler V, Naim M, Wikner J (2012). A control engineering approach to the assessment of supply chain resilience. International Journal of Production Research.

[CR72] Tabaklar T, Halldórsson Á, Kovács G, Spens K (2015). Borrowing theories in humanitarian supply chain management. Journal of Humanitarian Logistics and Supply Chain Management.

[CR73] Taleizadeh AA (2018). A constrained integrated imperfect manufacturing-inventory system with preventive maintenance and partial backordering. Annals of Operations Research.

[CR74] Thompson DDP, Anderson R (2021). The COVID-19 response: considerations for future humanitarian supply chain and logistics management research. Journal of Humanitarian Logistics and Supply Chain Management.

[CR75] Wamba SF, Akter S, Edwards A, Chopin G, Gnanzou D (2015). How ‘big data’ can make big impact: findings from a systematic review and a longitudinal case study. International Journal of Production Economics.

[CR76] Wang G, Gunasekaran A, Ngai EWT, Papadopoulos T (2016). Big data business analytics in logistics and supply chain management: certain investigations for research and applications. International Journal of Production Economics.

[CR77] Wong CWY, Wong CY, Boon-itt S (2018). How does sustainable development of supply chains make firms lean, green and profitable? A resource orchestration perspective. Business Strategy and the Environment.

[CR78] Yagci Sokat K, Altay N (2021). Serving vulnerable populations under the threat of epidemics and pandemics. Journal of Humanitarian Logistics and Supply Chain Management.

[CR79] Ye F, Liu K, Li LX, Lai KH, Zhan YZ, Kumar A (2022). Digital supply chain management in the COVID-19 crisis: An asset orchestration perspective. International Journal of Production Economics.

[CR80] Yu W, Jacobs MA, Chavez RJ, Yang J (2019). Dynamism, disruption orientation, and resilience in the supply chain and the impacts on financial performance: a dynamic capabilities perspective. International Journal of Production Economics.

[CR81] Yuen KF, Wang X, Ma F, Li KX (2020). The psychological causes of panic buying following a health crisis. International Journal of Environmental Research and Public Health.

[CR82] Zhao, J., Zhang, Y, He, X., and Xie, P. (2020). COVID-CT-Dataset: A CT scan dataset about COVID-19, 2020, arXiv preprint arXiv: http://arxiv.org/2003.13865.

